# GPX4-independent ferroptosis—a new strategy in disease’s therapy

**DOI:** 10.1038/s41420-022-01212-0

**Published:** 2022-10-30

**Authors:** Tianyu Ma, Jingtong Du, Yufeng Zhang, Yuyao Wang, Bingxuan Wang, Tianhong Zhang

**Affiliations:** 1 Department of Otorhinolaryngology Head and Neck surgery, The First Hospital affiliated to Harbin Medical University, Harbin, Heilongjiang China; 2 Department of Reproductive Endocrinology, The Second Hospital affiliated to Harbin Medical University, Harbin, China

**Keywords:** Prognostic markers, Molecular biology

## Abstract

Ferroptosis is a form of programmed cell death characterized by intracellular iron accumulation and lipid peroxidation, and earlier studies identified glutathione peroxidase 4 (GPX4) as an essential regulator of this process. Ferroptosis plays an essential role in tumors, degenerative diseases, and ischemia-reperfusion injury. However, researchers have found that inhibition of GPX4 does not entirely suppress ferroptosis in certain diseases, or cells express resistance to ferroptosis agonists that inhibit GPX4. As research progresses, it has been discovered that there are multiple regulatory pathways for ferroptosis that are independent of GPX4. The study of GPX4-independent ferroptosis pathways can better target ferroptosis to prevent and treat various diseases. Here, the currently inhibited pulmonary GPX4-dependent ferroptosis pathways will be reviewed.

## Facts

- Ferroptosis has been confirmed to play an important role in various biological processes, and GPX4 is a key enzyme.
- In the process of tumor treatment, inhibition of GPX4 is not always effective, and sometimes drugs resistances occur.
- Since the discovery of FSP1, more and more GPX4-independent ferroptosis pathways have been discovered in various physiological and pathological processes
- Drugs targeting GPX4-independent ferroptosis pathway may become a new direction for the treatment of diseases.

## Open questions

- How to promote ferroptosis in tumor cells and avoid ferroptosis in normal cells, thereby safely inhibiting tumor growth?
- Among all GPX4-independent ferroptosis pathways, are there common control factors that can regulate cellular ferroptosis?
- What factors are agonists of GPX4-independent ferroptosis?

## Background

The way of cell death has always been the center of people’s research. Since the discovery of apoptosis, people have known that cell death is a programmed process that follows specific regulatory laws. Other forms of cell death were discovered later, such as pyroptosis and necrosis. As a new form of programmed cell death, ferroptosis was first prescribed in 2012 [1]. Characterized by lipid peroxidation and abnormal iron accumulation, ferroptosis has attracted significant attention due to its unique association with pathological conditions such as ischemia-reperfusion injury, neurodegeneration, and cancer [2–4].

Glutathione peroxidase 4 (GPX4) acts as a master regulator in the ferroptosis process, and its unique function is to interrupt lipid peroxidation by converting lipid hydroperoxides into non-toxic lipid alcohols [5]. The biosynthesis of glutathione (GSH) and the normal function of GPX4 are central to the control of ferroptosis, and the inhibition of GPX4 can increase cellular ferroptosis sensitivity [6, 7]. Accumulation of iron produces specific phospholipid hydroperoxides, which are counteracted endogenously by cells through the system xc - /GSH/GPX4 axis. After cystine is absorbed by system xc, it will be reduced to cysteine to synthesize GSH, which maintains the activity of GPX4 [8, 9]. If any step of the process is disrupted, the activity of GPX4 will decrease, resulting in the accumulation of intracellular peroxides, which contributes to ferroptosis.

In the GPX4-dependent ferroptosis pathway, the Cap’n’collar (CNC) transcription factor nuclear factor erythroid-2 related factor 2 (NFE2L2/NRF2) plays an essential regulatory role in this process [10]. It can inhibit ferroptosis by activating GPX4 by activating SLC7A11. In addition, NRF2 is regulated by the cytoplasmic inhibitor Kelch-like ECH-associating protein 1 (KEAP1). KEAP1-mediated ubiquitination can target NRF2 for protein degradation. Under oxidative stress, the degradation of NRF2 by KEAP1 is activated [11].

A recent study found that NRF1 can also regulate GPX4 [12]. NRF1 is also a member of CNC transcription factors, which can promote the resistance of cells to ferroptosis by maintaining the expression of GPX4, independent of NRF2. Loss of GPX4 protein expression in NRF1-deficient cells sensitized cells to ferroptosis, whereas in cells lacking GPX4, overexpression of NRF1 failed to inhibit ferroptosis. Its regulation is also different from that of NRF2, which is mainly regulated by N-glycosylase 1 (NGLY1) and DNA loss inducer 1 homolog 2 (DDI2) [13, 14]. The functions of NRF1 and NRF2 pathways in development do not overlap, and their regulation of ferroptosis is independent.

The proto-oncogenic transcriptional co-activator YAP is also an important factor, which can inhibit ferroptosis upregulation of multiple ferroptosis modulators, including acyl-CoA synthetase long chain family member 4 (ACSL4) and transferrin receptor [15]. Activation of YAP can induce the expression of SLC7A11, thereby enhancing cystine uptake by System XC-, increasing GSH level, thereby increasing GPX4 synthesis, thus reducing ROS level and inhibiting ferroptosis [16, 17].

However, in the intervention process of ferroptosis, it was found that the application of GPX4 inhibitors could not completely block the occurrence of ferroptosis [18, 19], and some ferroptosis was also found in the process of tumour intervention. Tumours are resistant to ferroptosis agonists that inhibit GPX4 [20, 21], which suggests that there seem to be other ways that ferroptosis occurs. After continuous research by scientists, various new pathways of ferroptosis independent of GPX4 have been discovered. The discovery of these pathways can allow people to have more ways to increase or decrease the sensitivity of cells to ferroptosis. The application of ferroptosis is more promising, providing more possibilities for preventing and treating tumors, aging, and inflammation.

## NADPH-FSP1-CoQ10 pathway

In the study of the ferroptosis-resistant cell line MCF7, the researchers screened the complementary gene for GPX4 deletion and found the apoptosis-inducing factor mitochondrial 2 (AIFM2) gene, which was initially found to induce apoptosis, and the researchers named it ferroptosis suppressor protein 1 (FSP1) [22]. Since then, it has been proved that FSP1 can resist ferroptosis in some research [23–25].

Ferroptosis is driven by phospholipid peroxidation. Applying the GPX4 inhibitor RSL3 or knocking down GPX4 can enhance cell lipid peroxidation, while FSP1 overexpression attenuates RSL3-induced lipid peroxidation. Overexpression of FSP1 in GPX4 knockout cells can also significantly reduce phospholipid peroxidation products, thereby inhibiting the occurrence of ferroptosis, demonstrating that the anti-ferroptosis effect of FSP1 does not depend on the expression of GPX4. Previous studies have confirmed that the AIF family has NADH ubiquitin oxidoreductase activity [26], so FSP1 can regenerate lipophilic groups that capture oxygen radicals through NADPH. Reduced CoQ10 has an antioxidant effect by trapping oxygen free radicals in phospholipids and lipoproteins [27]. The researchers generated CoQ10-deficient HT1080 cells by deleting 4-hydroxybenzoate poly pentenyl transferase (COQ2) that catalyzes the first step in CoQ10 biosynthesis. Overexpression of FSP1 effectively inhibited ferroptosis in HT1080 cells but failed to inhibit ferroptosis in COQ2-deficient cells, which indicates that the anti-ferroptosis effect of FSP1 is dependent on CoQ10. It was also found that the Michaelis constant (Km = 1.2 × 10 − 5 M) of FSP1 was relatively low, and the maximum reaction rate (Vmax = 4.1 × 10 − 7 M s − 1) was much higher than that of related oxidoreductases, indicating that efficiency of FSP1. In another study, the researchers found that the expression of FSP1in lung cancer enhances the resistance of cancer cells to ferroptosis. The myristoylated FSP1 can be recruited into the plasma membrane, inhibiting ferroptosis through the antioxidant effect of CoQ10, generating a lipophilic radical trapping antioxidant (RTA) that halts the propagation of lipid peroxides [23].

Currently, the regulatory mechanism of FSP1 in cells is unclear, but exogenous miRNAs can regulate it. Tumor cells can release exosomes around to regulate the outside world, which is beneficial to their survival and enhances resistance to chemotherapy drugs [28]. It has been found that cancer cells can activate FSP1-mediated ferroptosis resistance in surrounding tumor cells by secreting miRNAs through exosomes. Song’s research on non-small cell lung cancer (NSCLC) found that there is ferroptosis in cancer cells treated with cisplatin, and ferroptosis-resistant cancer cells could secrete miR-4443 through exosomes, which increase the expression of FSP1 in ferroptosis-sensitive cancer cells, thereby enhancing the resistance of the cancer cells to ferroptosis [29]. Methyltransferase-like 3(METTL3) is a methyltransferase that can achieve M6A methylation, which can inhibit the expression of FSP1. Bioinformatics analysis found that METTL3 was a target gene of miR4443. A 2-fold reduction in M6A content was found in cells overexpressing miR-4443, whereas the expression of FSP1 was upregulated.

These findings suggest that pharmacological inhibition of FSP1 may provide an effective strategy for cancer therapy by sensitizing cancer cells to ferroptosis-inducing chemotherapeutic drugs and, if used in combination with GPX4 inhibitors, could have a strong synergistic effect.

## P53-mediated lipoxygenase and iPLA2β pathway

As a tumor suppressor, p53 can inhibit tumor cells by arresting the cell cycle, promoting apoptosis and senescence [30, 31]. In addition, studies have found that p53 plays a vital role in regulating the ferroptosis of cancer cells through its metabolic targets. The accumulation of intracellular ROS caused by low levels of tert-butyl hydroperoxide can induce the activation of p53 and lead to cell death, which can be specifically inhibited by the ferroptosis inhibitor Fer-1 but cannot be inhibited by other kinds of cell death pathway inhibitors, indicating that the form of death is ferroptosis. P53 can inhibit cystine uptake by inhibiting the expression of SLC7A11, a vital component of the cystine/glutamate reverse transporter, thereby enhancing the sensitivity of cells to ferroptosis [32]. However, Chu found that activation of p53 alone did not significantly downregulate GSH/GSSG ratio, glutathione levels, and GPX4 activity in human cancer cells. Although p53-induced downregulation of SLC7A11 could partially inhibit cystine uptake, the overall effect on GSH/GSSG ratio and glutathione levels was less. However, the researchers found that intracellular lipoxygenase levels were significantly increased. So P53 may act on reactive oxygen species (ROS)-induced stress through lipoxygenase [19].

The mammalian lipoxygenase family includes six distinct isoforms (ALOXE3, ALOX5, ALOX12, ALOX12B, ALOX15, and ALOX15B), and the researchers found that only the absence of ALOX12 significantly reduced ferroptosis. However, when ALOX12 was depleted, the expression of p53 and SLC7A11 was not affected [19]. The ALOX12 gene is located on human chromosome 17p13.1, a common location for monoallelic deletions in cancers. Under the harsh conditions of cell survival, lipoxygenase can oxidize lipids, resulting in lipid peroxidation. The loss of ALOX12 inhibits p53-mediated ferroptosis and accelerates tumor progression. Acyl-CoA synthase long-chain family member 4 (ACLS4) is a fatty acid activating an enzyme that catalyzes the esterification of long-chain PUFAs such as arachidonic (AA) and adre-noyl (AdA) to form PE, which is an integral component in the GPX4-dependent ferroptosis pathway [33, 34]. ACSL4 is required to inhibit GPX4-induced ferroptosis, while ALOX12 is dispensable, whereas not required in p53-mediated ferroptosis, and therefore p53 promotes ferroptosis through a GPX4-independent pathway. Notably, deletion of GPX4 caused severe phenotypic abnormalities in mice in animal experiments, whereas deletion of the ALOX12 gene did not cause major developmental defects in mice. Wang found that in RSL3-induced ferroptosis, both the protein expression and acetylation level of ALOX12 were significantly increased, thereby aggravating intracellular phospholipid peroxidation [35]. At present, relevant studies have confirmed that the expression of ALOX12 can reasonably predict the prognosis of patients, and ALOX12 can be used as a predictor of tumor prognosis [36–38].

In addition, iPLA2β is a significant inhibitor of p53-mediated ferroptosis under ROS-induced stress. The phospholipase A2 (PLA2) gene family encodes proteases that specifically hydrolyze ester bonds on phospholipids to generate free fatty acids and lysophosphatidic acid [39]. Calcium-independent phospholipase A2β (iPLA2β), a member of the PLA2 family, is a cytoplasmic protein with catalytic activity in the absence of calcium, and previous studies have shown that it is associated with metabolic disorders and inflammation [40]. Chen found in human melanoma cells that silencing iPLA2β would enhance the sensitivity to p53-mediated ferroptosis without significant effects on cells that usually express iPLA2β, suggesting that iPLA2β can inhibit p53-mediated ferroptosis, and p53-driven ferroptosis is independent of GPX4 [41]. The same results were found in osteosarcoma cells and breast cancer cells. Studies have shown that the PUFA containing phospholipids is the key to intracellular ferroptosis. When cells are under external stress, iPLA2β can hydrolyze oxidized PUFA-PLs to inhibit p53-induced ferroptosis. Sun found that 15-HpETE-PE is a preferential recognition substrate of iPLA2β, and iPLA2β can be hydrolyzed, inhibiting cell ferroptosis [42]. They found that the onset of Parkinson’s was closely related to ferroptosis. R747W is a mutant phenotype with loss-of-function of the gene encoding iPLA2β, which reduces the hydrolytic activity of 15-HpETE-PE, and increases the content of 15-HpETE-PE in cells, and increases the sensitivity to ferroptosis compared with the wild type. Beharier found that spontaneous preterm birth associated with placental dysfunction showed placental ferroptosis, accumulation of lipid peroxides in the placenta, and increased sensitivity of iPLA2β-deficient trophoblast cells to ferroptosis [43]. Inactivation of iPLA2β in BeWo cells significantly enhanced ferroptosis induction by RSL3. After silencing GPX4 expression, knockdown of iPLA2β enhanced the sensitivity of cells to ferroptosis.

ALOX12 is essential for p53-mediated ferroptosis, and in ALOX12-overexpressing cells, there were significantly elevated levels of oxidized lipids. After adding the iPLA2β overexpression vector, the elevated lipid peroxidation was effectively reduced, and the ferroptosis of cells was inhibited at the same time. These suggest that iPLA2β can inhibit ferroptosis by eliminating ALOX12-induced lipid peroxidation.

## iNOs/NO pathway

It has been found that ferroptosis plays a role in different mammalian cells, but some specific types of cells, especially cells of the innate immune system (such as macrophages), show significant resistance to ferroptosis [44]. M1-type macrophages are resistant to ferroptosis, while M2 and M0-type macrophages show sensitivity. In the exploration of the known pathways of cell resistance to ferroptosis, the contents of 15-LOX, ACSL4, and LPCAT3 did not differ significantly, indicating that the ferroptosis resistance of M1 macrophages does not rely on traditional pathways. After further testing, the researchers found that the contents of nitric oxide synthase (iNOS) and NO in M1 macrophages were significantly higher than those in M0 and M2 types. After the inactivation of iNOS, M1 macrophages were less resistant to ferroptosis [45], while after transfection of the iNOS plasmid into M2 macrophages, their ferroptosis resistance was significantly enhanced. By lipidomic measurement, the content of lipid peroxides in cells was significantly reduced after transfection of the iNOS plasmid.

This mechanism plays an important role in host resistance to bacterial infection. After the bacterium infects the host cell, the host cell and the bacterium will work for their favorable living environment. The host employs a variety of programmed cell death to fight infection, while the bacteria regulate the cell death program. Recently, it was found that the Gram-negative bacterium Pseudomonas aeruginosa (PA) can lead to the loss of intracellular GPX4 after infection of the host, resulting in ferroptosis [46]. In mammalian cells, the PA can release the lipoxygenase and improve the level of cell lipid oxidation after infection. In addition, the degradation of intracellular GPX4 through CMA pathways can lead to the accumulation of intracellular phospholipid peroxide and ferroptosis, and host cells usually use GPX4 for peroxide phospholipids reduction, thus protecting cells from death. Dar found that when PA infected epithelial cells, M1-activated macrophages expressed iNOS/NO in the host immune cells, which could make the cells resistant to ferroptosis [47]. NO is an active molecule produced by the nitric oxide synthase (NOS) protein family, which directly binds and inactivates iron-containing enzymes [48] or reacts with superoxide anion radical O2 to form a highly active peroxy sulfoxide Nitrates (ooo-), which can attack membrane lipids and proteins of the pathogens, especially protein thiols [49]. In this way, NO exerts a bactericidal effect on macrophages. At the same time, the researchers found that lung epithelial cells were surrounded by host immune cells, and the number of macrophages did not increase before and after infection, while the expression level of iNOS increased. Macrophages significantly attenuated the lipid peroxidation of epithelial cells. This study confirmed that macrophages secreted NO to adjacent epithelial cells, preventing phospholipid peroxidation in epithelial cells, thereby inhibiting ferroptosis, while NO expression did not affect intracellular GPX4 levels. After knockdown the GPX4 gene in epithelial cells, the researchers found that NO inhibits ferroptosis in epithelial cells even when GPX4 is deficient. Further studies have found that when intracellular iron and lipid disorders are disturbed, 15-hydroperoxy-arachidonoyl-phosphatidyl-ethyl amino alcohol (15-HpETE-PE) will accumulate, resulting in the appearance of ferroptosis [4, 34, 50], and one of the catalysts of this reaction may be the ALOX15 and scaffold protein PE-binding protein-1 (PEBP1) complex [51, 52], NO can inhibit the catalysis of this complex, thereby inhibiting ferroptosis.

Pseudomonas aeruginosa is an opportunistic pathogen that can quickly multiply when the body is immunocompromised. Some studies have found that the abundance of PA significantly increased in the radiotherapy of cancer patients. If NO therapy can be introduced in these patients, it may become a promising new treatment strategy.

## DHODH

Dihydroorotate dehydrogenase (DHODH) is a flavin-dependent mitochondrial enzyme that catalyzes the fourth step in the de novo synthesis of pyrimidine nucleotides and is a key enzyme in pyrimidine synthesis. DHODH is an enzyme located on the outer surface of the inner mitochondrial membrane [53], so it only functions in mitochondria. DHODH catalyzes the conversion of dihydrohexoacetate to hexoacetate through REDOX reaction [54]. Then, the supine acid is converted to uridine monophosphate, and RNA is involved in ribosome biogenesis. Initially used to treat rheumatoid arthritis and multiple sclerosis, DHODH inhibitors were later found helpful in treating cancer, viral infections, and blood disorders [55–58]. Recent studies have found that it also plays a role in ferroptosis. Mao found that treating cancer cells with GPX4 inhibitors resulted in acute depletion of N-carbamyl-L-aspartate (C-ASP, a pyrimidine biosynthesis intermediate), accompanied by accumulation of uridine, which could be inhibited by fer-1, a ferroptosis inhibitor [18]. Supplementation with DHO or OA(substrates and products of DHODH) attenuated and enhanced GPX4-induced iron death, respectively, especially in cancer cells with low GPX4 expression. After supplementation with DHO or OA (the substrate and product of DHODH), the ferroptosis caused by GPX4 inhibition was attenuated and enhanced, respectively, and these effects were particularly significant in cancer cells with low GPX4 expression. The substrates and products of the DHODH reaction have opposite effects on ferroptosis, suggesting that the potential role of DHODH in regulating ferroptosis sensitivity is independent of its function of producing pyrimidine nucleotides. Inactivation of DHODH in cancer cells with low GPX4 expression significantly increased intracellular lipid peroxidation, while in cancer cells with high GPX4 expression, DHODH inactivation synergized with inducers to enhance ferroptosis.

There are two types of GPX4 localization in mammalian cells: cytoplasm and mitochondria. The inhibition of DHODH only affects the changes of GPX4 in mitochondria. FIN56, a ferroptosis inducer, mainly induces ferroptosis by depleting GPX4 protein and ubiquitin ketone CoQ [59], which can induce mitochondrial lipid peroxidation. Inhibition of DHODH cannot increase the sensitivity of cells to FIN56-induced ferroptosis, while the decrease in CoQ cells was more sensitive to ferroptosis than DHODH knockout cells. Therefore, the anti-ferroptosis function of DHODH is dependent on CoQ. In mitochondria, DHODH can reduce CoQ to ubiquinol (CoQH2), reducing oxygen free radicals, inhibiting the generation of phospholipid hydroperoxide (PLOOH), and inhibiting phospholipid peroxidation, thereby inhibiting ferroptosis. DHODH inhibitor treatment cannot affect the expression of GPX4, SLC7A11 or ACSL4, or GSH levels.

GPX4 and DHODH constitute the two primary defense weapons against lipid peroxidation in mitochondria. Inactivation of one pathway can compensate for the other pathway, and both pathways inactivated can trigger mitochondrial lipid peroxidation. These data suggest that DHODH inhibition induces ferroptosis in GPX4-low cancer cells while sensitizing GPX4-high cancer cells to ferroptosis. Sulfasalazine can inhibit system xc, so the combination of DHODH inhibitors and sulfasalazine may become a new strategy for cancer treatment.

## GCH1/BH4 pathway

Mammalian cells have evolved different mechanisms to cope with increased levels of oxidative damage. One metabolic pathway frequently involved in reactive oxygen species production includes GCH1/tetrahydrobiopterin (BH4), where GCH1 is the rate-limiting enzyme of BH4 synthesis. BH4 is an essential cofactor for producing aromatic amino acids, neurotransmitters, and nitric oxide in the presence of iron at the enzyme-catalyzed site. The loss of BH4 leads to the decoupling of nitric oxide synthase (NOSs) and superoxide formation. GCH1 is involved in various diseases, including pain sensitivity [60] and some chronic diseases, such as hypertension and diabetes [61, 62]. Some studies also found its role in cancer [63, 64]. In addition, some studies have confirmed that GCH1/BH4 plays a vital role in ferroptosis. By administering the ferroptosis inducers RSL3 and IKE and knocking out the GPX4 gene in immortalized mouse fibroblasts, respectively, Kraft identified a common gene, GCH1, in the surviving cells by genetic sequencing [65]. GCH1 can effectively rescue ferroptosis induced by ferroptosis inducers and GPX4 deletion, but not apoptosis and necrosis. Metabolic analysis revealed that the source of the anti-ferroptosis effect of GCH1-overexpressing cells was BH4/BH2, supplementation with which rescued cell viability. In addition, in cultured cells, overexpression or underexpression of GCH1 made cancer cells correspondingly resistant or sensitized to ferroptosis. It was found that the expression of GCH1 triggers the production of potent antioxidants BH4/BH2, which can block lipid peroxidation and ferroptosis, whether supplemented exogenously or produced endogenously. These potent metabolites have antioxidant capacity comparable to or greater than those of the ferroptosis inhibitors Lip-1 and Fer-1.

One pathway by which BH4 inhibits ferroptosis is by inducing plasma membrane lipid remodeling. Non-targeted lipidomic analysis revealed that levels of lysophosphatidylcholines (LysoPCs) and lysophosphatidylethanolamines (LysoPEs) were significantly increased in both GCH1-expressing and overexpressing cells following IKE treatment and contained PUFAs. The phospholipids of the chains are extensively consumed because the accumulation of lysophospholipids results from the degradation of the oxidized PUFA tails by enzymatic cleavage. Both triacylglycerol (TAG) and diacylglycerol (DAG) present in lipid droplets are depleted, containing PUFA tails. These changes suggest that GCH1 overexpression does not protect all PUFA-PLs, but selectively blocks the oxidation of specific PUFA-PLs. It was found that GCH1 overexpression selectively protected phosphatidylcholines with two PUFA tails from oxidative degradation, and these phospholipids drove ferroptosis, thereby protecting cells from ferroptosis. GCH1 can also protect cells against oxidative damage by protecting CoQ10. The content of CoQ10 significantly increased in GCH1-overexpressing cells, possibly due to the abundant production of its biosynthetic precursor 4-OH-benzoate, which is dependent on BH4-mediated conversion of phenylalanine to tyrosine. Under oxidative stress, cells with high BH4 levels can synthesize CoQ10 to alleviate oxidative damage. Hu [20] found that many colon cancer (CRC) cell lines were resistant to ferroptosis induced by the traditional ferroptosis inducers erastin and RSL3. They demonstrated that BH4 determines the sensitivity of CRC cells to erastin-induced ferroptosis. Inhibition of GCH1 reduced BH4 and resulted in enhanced erastin-induced iron accumulation and lipid peroxidation, thereby enhancing ferroptosis sensitivity, while BH4 supplementation suppressed ferroptosis-related changes caused by GCH1 downregulation. Knockdown of GCH1 significantly activated ferritin autophagy upon erastin treatment, which was reversed by using an autophagy inhibitor. The results show that the combination of GCH1 inhibitor and erastin can synergistically inhibit the growth of cancer cells. In Gpx4 knockout cells, GCH1 overexpression can significantly protect cells from ferroptosis, and GPX4 and glutathione levels are not affected by GCH1 overexpression. GCH1 protects cells from ferroptosis mainly through the antioxidant effects of BH4/BH2, utterly independent of gpx4-mediated ferroptosis.

## Ferritin and prominin2

Ferritin, consisting of 24 subunits, is the major intracellular iron storage protein complex, including ferritin light polypeptide 1 (FTL1) and ferritin heavy polypeptide 1 (FTH1). Increased ferritin expression can reduce the iron level in the body, thereby limiting ferroptosis. Studies have indicated that the increase of the autophagy level in vivo can lead to the degradation of ferritin and the increase of iron level, thus leading to oxidative damage of cells through the Fenton reaction [66, 67]. Autophagy-related genes (Atg) play a role in this process, among which Atg5 and Atg7 are crucial for the formation of autophagosomes [68]. In Atg5-deficient cells, erastin-induced ferroptosis is inhibited, and ferritin levels are significantly elevated, suggesting that Atg5 mediates ferritin autophagy. In this process, as a selective receptor for ferritin autophagy, the level of NCOA4 also affects the level of ferritin. In NCOA4 knockout cells, the content of Fe2+ is reduced, which leads to the inhibition of ferroptosis. Fuhrmann found that the content of NCOA4 in macrophages decreased under hypoxic conditions, increasing intracellular ferritin content, and thereby enhancing macrophage resistance to ferroptosis [69].

The relationship between autophagy and ferroptosis is still controversial, and it is widely accepted that autophagy induces ferroptosis. A recent study found that the ferroptosis inducer erastin can induce cellular autophagy, and autophagy can trigger ferroptosis by degrading ferritin and transferrin receptor 1 (TfR1), resulting in the accumulation of intracellular iron. In autophagy-deficient cells, intracellular iron and lipid peroxidation were decreased, resulting in cells that could become resistant to erastin-induced ferroptosis [70]. Li’s research indicated that lipopolysaccharide (LPS) induced cardiac ferroptosis in mice with sepsis. LPS can increase the expression of NCOA4 and enhance ferritin autophagy, leading to increased intracellular iron and increased ferroptosis. Therefore, ferritin autophagy-induced ferroptosis is one of the critical mechanisms of sepsis-induced cardiac injury [71]. Fang found that in the myocardium of mice specifically deficient in ferritin H (Fth), iron levels decreased, and oxidation levels increased, resulting in mild cardiac aging damage. Severe cardiac damage occurred when given these mice a high-iron diet and hypertrophic cardiomyopathy, with typical features of ferroptosis, which were rescued by administration of Fer-1 [72]. Rui found that in traumatic brain injury, melatonin, as a primary hormone of the pineal gland, plays a vital role in brain protection after traumatic brain injury [73]. Relying on melatonin receptor 1B (MT2) can protect neurons, but in Fth-specific knockout mice, melatonin protection was essentially abolished.

Prominin is a plasma membrane glycoprotein specifically associated with plasma membrane protrusions of epithelial and non-epithelial cells (neural and hematopoietic stem cells). Prominin 2 has a typical membrane topology with five transmembrane segments and two large glycosylated extracellular loops [74]. Brown found that the separation of breast cancer cells from the extracellular matrix (ECM) significantly decreased cell viability, whereas cell viability was improved by the ferroptosis inhibitor Fer-1, demonstrating that separation of cells from the ECM is a pro-ferroptosis response. The expression of prominin 2 and the gene PROM2 encoding prominin 2 significantly increased in cells that survived under this condition, indicating that prominin 2 can inhibit cell ferroptosis. Further study found that the expression of exogenous prominin 2 inhibited cell ferroptosis induced by RSL3, ML210, FIN56, and erastin. Immunofluorescence showed that prominin 2 co-localized with markers of multivesicular bodies (MVBs) within 30 min after GPX4 inhibition, most evident in the perinuclear region. Adding exogenous prominin 2 to prominin-deficient cells can increase the content of MVBs, confirming that the formation of MVBs is dependent on prominin 2. MVBs can fuse with the plasma membrane and release intraluminal vesicles to form exosomes. Exosome formation increased after GPX4 inhibition, which contained prominin and TSG101, as well as ferritin, while blocking the export of exosomes resulted in the accumulation of iron, indicating that in the state of cellular oxidative stress, prominin 2 can promote the formation of iron-containing exosomes and transport intracellular iron to the extracellular space, thereby enhancing cell resistance to ferroptosis [75]. There is currently no specific inhibitor of prominin2, so it is impossible to inhibit prominin2 directly. However, in the study, it was found that 4-hydroxynonenal (4HNE) is a lipid metabolite formed from lipid peroxidation products. Under the mediation of p38MAP kinase, heat shock factor 1 (HSF1)-dependent activation of PROM2 transcription can be inhibited by the application of HSF1 inhibitors. A combination of ferroptosis inducers and HSF1 inhibitors resulted in tumor growth arrest in mice. That provided a new idea for treating ferroptosis-resistant tumors [76].

## SREBP1-SCD1-MUFA pathway

Ferroptosis is characterized by lipid peroxidation, so the type of fatty acid can significantly influence it. Polyunsaturated fatty acids (PUFAs) are the main components of biofilms, and cells with high PUFA levels are more sensitive to ferroptosis. Zou found that peroxisomes promoted lipid peroxidation by synthesizing polyunsaturated ether phospholipids (PUFA-ePLs), which acted as substrates for lipid peroxidation, leading to ferroptosis [77]. Monounsaturated fatty acid (MUFA) is another component in the biofilm, which is non-oxidizable, and its increase can reduce the content of PUFA. Therefore, cells with high MUFA content are more resistant to ferroptosis, and MUFA can make cells ferroptosis insensitive [78]. Exogenous MUFA can reduce lipid ROS accumulation and inhibit ferroptosis in an acyl-CoA synthase long-chain family member 3 (ACSL3)-dependent manner. ACSL3 can convert MUFAs to MUFA-pl, replacing PUFAs in the plasma membrane and alleviating ROS accumulation [79].

It has been found that MUFA is regulated by SREBP1-SCD1 in vivo. The sterol regulatory element-binding protein (SREBP) family of transcription factors plays a crucial role in lipid metabolism by regulating the expression of a series of enzymes required for lipid synthesis, and nuclear fragments of transcriptionally active SREBPs (nSREBPs) are unstable, degraded by the ubiquitin-proteasome pathway. SREBP1a and SREBP1c are preferentially involved in fatty acid biosynthesis, while SREBP2 mainly regulates genes in the cholesterol biosynthesis pathway [80]. Stearoyl-CoA desaturase 1 (SCD1) is an iron-containing endoplasmic reticulum-binding enzyme that can modify lipids and play an essential role in synthesizing saturated fatty acids (SFAs) and MUFAs. SCD1 is a downstream gene of SREBP1 and is regulated by it. Yi found that the canonical pathway PI3K-AKT-mTOR can increase cellular resistance to ferroptosis [81]. This pathway is activated when PI3K mutations or phosphatase loss and tensin homolog deletion on chromosome 10 (PTEN) function. This pathway mainly regulates ferroptosis by inducing and activating the expression of SREBP1, which can target and activate the downstream SCD1, and SCD1 can inhibit cell ferroptosis by increasing the content of MUFA. Tesfay found that in ovarian cancer stem cells, inhibition of SCD1 expression led to ferroptosis in cancer cells. After overexpression of SCD1, the researchers found that lipid peroxides significantly reduced, consistent with the results of exogenous MUFA supplementation [82]. The researchers found that SCD1 can catalyze the formation of monounsaturated fatty acids from saturated fatty acid acyl CoA. Inhibiting SCD1 reduces the content of MUFA and increases the content of PUFA, which can provide a corresponding substrate for lipid peroxidation.

## Summary and prospect

**Fig. 1:**
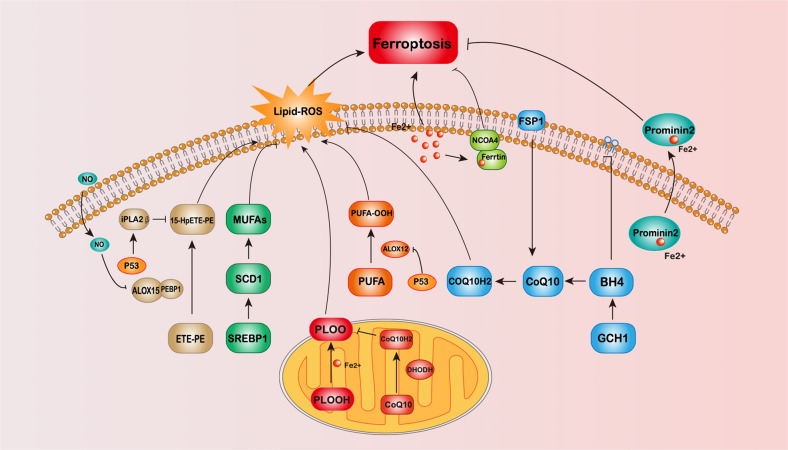
Schematic diagram of the GPX4-independent ferroptosis pathway. The figure shows 7 pathways of GPX4-independent ferroptosis pathway mentioned in the review, including NADPH-FSP1-CoQ10 pathway, P53-mediated lipoxygenase, and iPLA2β pathway, iNOs/NO pathway, DHODH pathway, GCH1/BH4 pathway, Ferritin, and prominin2 pathway and SREBP1-SCD1-MUFA pathway.

With the discovery that FSP1 can regulate ferroptosis independent of GPX4, researchers have become more enthusiastic about finding GPX4-independent ferroptosis pathways. ALOX and iPLA2β, iNOS, GCH1, DHODH, Prominin2, MUFA, and other regulatory pathways regulated by FSP1 and p53 have been found (Fig. 1). With more and more ferroptosis regulatory pathways discovered, drugs or genetic manipulations targeting these pathways have been applied to treat diseases with positive results. Nevertheless, there are still many questions to be answered in the study of ferroptosis.

Firstly, the determination of ferroptosis, often based on cellular responses to ferroptosis inducers or ferroptosis inhibitors, are antioxidants in nature and may inhibit other ROS-dependent cell death. There is no clear diagnostic marker that can directly prompt ferroptosis and quantitative analysis of ferroptosis. Secondly, the present research has not clarified how cells die during ferroptosis. We have identified iron accumulation and lipid peroxidation during this process. However, this is an intermediate process, not the final result, and the mechanism behind the final cell death is still unclear. Does membrane fluidity become restricted, leading to the failure of membrane-related functions, or does the structural disruption of the membrane lead to ferroptosis due to membrane rupture? It is an issue that future research needs to address. Finally, in most forms of cell death, there is the accumulation of oxygen free radicals, mitochondrial dysfunction, et al. The regulatory factors of ferroptosis and other forms of cell death also overlap. That is to say, the factors that regulate ferroptosis can also regulate cells. In processes such as apoptosis and necrosis, how cells choose to die remains a mystery.

With the deepening of ferroptosis research, its function in cell survival has been continuously discovered. Continuous exploration of its mechanism can allow people to understand ferroptosis better and utilize iron better. Death to achieve the purpose of gaining health and prolonging life. Researchers will also continue to concentrate on research to discover more secrets of iron death.

## Data Availability

Data sharing is not applicable to this article as no new data were created or analyzed in this study.
